# 

**Published:** 1936

**Authors:** 


### The Medical Library of the University of Bristol,

WITH WHICH IS INCORPORATED

### The Library of the Bristol Medico-Chirurgical Society.

---

*The following donations have been received since the publication of the list in March,* 1936.

June, 1936.

| | |
|---|---|
| American Laryngological, Rhinological and Otological Society (1) .. .. .. .. | 1 volume. |
| Messrs. Bayer Products (2) .. .. .. | 1 ,, |
| British Dental Association (3) .. .. .. | 1 ,, |
| Professor R. J. Brocklehurst (4) .. .. | 1 ,, |
| Buenos Aires University (5) .. .. .. | 1 ,, |
| California University (6) .. .. .. .. | 1 ,, |
| "Éditions Biodynamisme" (7) .. .. .. | 1 ,, |
| General Medical Council (8) .. .. .. | 3 volumes. |
| Professor E. W. Hey Groves (9) .. .. | 6 ,, |
| Professor Dr. C. Robertson Lavalle (10) .. .. | 1 volume. |
| J. E. R. McDonagh, Esq. (11) .. .. .. | 1 ,, |
| A. Cecil Powell, Esq. (12) .. .. .. | 1 ,, |
| Dr. J. Odery Symes (13) .. .. .. | 1 ,, |

Unbound periodicals have also been received from Professor E. W. Hey Groves, Professor C. Bruce Perry, Dr. J. Odery Symes, and Dr. T. Milling.

### THE ONE HUNDRED AND FIFTY-FIFTH LIST OF BOOKS.

The figures in round brackets refer to the figures after the names of the donors. The books to which no such figures are attached have either been bought from the Library Fund or received through the *Journal.*

**Allen, B. M.** .. .. *Glands and Growth* [Faculty Research Lecture at the University of California at Los Angeles] .. .. .. .. .. .. .. (6) 1930

**Donaldson, M., and others.** *The Early Diagnosis of Malignant Disease* .. .. .. .. .. .. .. .. .. 1936

**Estripeaut, R.** .. *Études sur l'Étiologie du Cancer* .. .. (7) 1936

**Franz, C.** .. .. .. *Lehrbuch der Kriegschirurgie* .. .. .. (9) 1936

**French, H. (ed.)** .. *Index of Differential Diagnosis of Main Symptoms* .. .. .. .. .. 5th Ed. (9) 1936

**Golden, R. (ed.)** .. *Diagnostic Roentgenology* .. .. .. (9) 1936

**Henderson, D. K., and Gillespie, R. D.** *A Text-Book of Psychiatry* 4th Ed. 1936

**Hill, D., and Howitt, F.** *Insulin: Its Production, Purification and Physiological Action* .. .. .. .. .. .. 1936

**Hodson, C. B. S.** .. *Human Sterilization To-day* .. .. .. (13) 1934

**Lavalle, Dr. C. R.** .. *Coxalgia: Tratamiento Quirurgico* .. (10) 1934

*Libro de Oro del Profesor Don Enrique Finochietto* .. .. (5) 1935

**McDonagh, J. E. R.** .. *The Common Cold and Influenza* .. (11) 1936

**Maingot, R. (ed.)** .. *Post-Graduate Surgery.* Vol. II. .. .. (9) 1936

*Medicine in its Chemical Aspects.* Vol. II. .. .. .. .. .. (2) 1933

**Orr, J. B.** .. .. *Food, Health and Income* .. .. .. .. .. 1936

**Pauwels, F.** .. .. *Der Schenkelhalsbruch* .. .. .. .. .. (9) 1935

**Roberts, Ff.** .. .. *Principles and Practice of X-ray Therapy* .. 1936

**Roche, A. E.** .. .. *An Anthology of Wit* .. .. .. .. .. .. 1935

**Weeks, C. C.** .. .. *Alcohol in Hospital Practice* .. .. .. .. 1936

**Williams, G.** .. .. *Minor Surgery and the Treatment of Fractures* 1936

21st Ed. (9) 1936

**Woods, R. R.** .. .. *Painful and Dangerous Diseases of the Ear* .. 1936

**Wright, S.** .. .. *Applied Physiology* .. .. .. 5th Ed. (4) 1934

### TRANSACTIONS, REPORTS, JOURNALS, ETC.

British Dental Association List of Members .. .. .. .. .. (3) 1936

British Journal of Surgery. Vol. XXIII, No. 92. April, 1936 .. 1936

Collected Papers of the Mayo Clinic and the Mayo Foundation, XXVII 1935

Dentists' Register .. .. .. .. .. .. .. .. .. .. .. .. .. (8) 1936

Medical Annual .. .. .. .. .. .. .. .. .. .. .. .. .. .. 1936

Medical and Dental Students' Register.. .. .. .. .. .. .. (8) 1935

Medical Register .. .. .. .. .. .. .. .. .. .. .. .. .. (8) 1936

Microscopical Journal. Vol. I, II. 1 vol. .. .. .. .. .. (12) 1869

Quarterly Cumulative Index Medicus. Vol. 18 (July-December) .. 1935

Surgical Clinics of North America. April, 1936 .. .. .. .. .. 1936

Transactions . . . of the American Laryngological Association 1933, 1934, 1935

Transactions of the Forty-first Annual Meeting of the American Laryngological, Rhinological and Otological Society .. .. (1) 1935

### Publications Received.

From J. W. Arrowsmith Ltd. :

*William Budd* . . . . By E. W. Goodall, O.B.E., M.D. Price 5s.

From Baillière, Tindall and Cox :

*Synthetic Anatomy.* By J. E. Cheesman. Complete in 14 parts. 3s. per part.

From John Bale, Sons and Danielsson :

*Narrative of an Investigation concerning an Ancient Medicinal Remedy and its Modern Utilities* . . . , by C. J. MacAlister, M.D., F.R.C.P., together with *An Account of the Chemical Constitution of Allantoin*, by A. W. Titherley, D.Sc., Ph.D. Price 2s. 6d.

From Cambridge University Press :

*Johannes de Mirfeld, of St. Bartholomew's Hospital, Smithfield : his Life and Works.* By Sir Percival Horton-Smith Hartley, C.V.O., M.A., M.D., F.R.C.P., and Harold Richard Aldridge, M.A. Price 15s.

From Cassell & Co. :

*The Vegetative Nervous System.* By Wulf Sachs, M.D., with an introduction by Sir Walter Langdon Brown, M.D., F.R.C.P. Price 15s.

From J. & A. Churchill :

*The Relief of Pain* . . . . By Harold Balme, M.D., F.R.C.S., D.P.H. Price 12s. 6d.

From William Heinemann Ltd. :

*The Common Cold and Influenza : the Nature of Disease Annual Reports for the years 1934 and 1935.* By J. E. R. McDonagh, F.R.C.S. Price 12s. 6d.

*Vitamins and other Dietary Essentials.* By W. R. Aykroyd, M.D. 2nd Edition. Price 7s. 6d.

From H. K. Lewis & Co. :

*Treatment in General Practice* . . . . Republished from *The British Medical Journal.* Price 8s. 6d.

*Vitality and Energy in Relation to the Constitution.* By T. E. Hammond, F.R.C.S. Price 12s. 6d.

*Nature and Treatment of Asthma, Hay Fever and Migraine* . . . . By Alexr. Gunn Auld, M.D. Price 12s. 6d.

*The Prevention of Cancer.* By W. Sampson Handley, M.S., F.R.C.S. Price 1s.

*A Short Practice of Surgery.* By H. Bailey, F.R.C.S., and R. J. McNeill Love, M.S., F.R.C.S. 3rd Edition. Price 28s.

From Macmillan & Co. :

*Vascular Disorders of the Limbs.* By Sir Thomas Lewis, F.R.S., M.D., D.Sc., LL.D., F.R.C.P. Price 6s. 6d.

From Methuen and Co. :

*Hero Dust.* By James Kemble, Ch.M., F.R.C.S. Price 6s.

From THE NATIONAL TEMPERANCE LEAGUE :

*Alcohol in Hospital Practice.* By COURTENAY C. WEEKS, M.R.C.S., L.R.C.P. Price 9d.

From OXFORD UNIVERSITY PRESS :

*The Anaemias.* By JANET M. VAUGHAN, D.M., M.R.C.P. 2nd Edition. Price 12s. 6d.

*Applied Physiology.* By SAMSON WRIGHT, M.D., F.R.C.P. 6th Edition. Price 20s.

*Squint Training.* By M. A. PUGH, M.R.C.S., L.R.C.P. Price 7s. 6d.

*The Endocrine Organs in Health and Disease.* By Sir HUMPHRY DAVY ROLLESTON, Bart., G.C.V.O., K.C.B., M.D., D.Sc., D.C.L., LL.D. Price 36s.

From THE STERLING MEDICAL PUBLISHING CO. :

*Aural Therapy in Relation to Deafness.* By Professor D. F. FRASER-HARRIS, M.D., D.Sc., B.Sc. (Lond.), F.R.S.E., with a foreword by Sir JAMES PURVES-STEWART. 2nd Edition. Price 7s. 6d.

From JOHN WRIGHT & SONS :

*Symptoms and Signs in Clinical Medicine* . . . . By E. NOBLE CHAMBERLAIN, M.D., M.Sc., M.R.C.P. Price 25s.

*A Manual of Practical Obstetrics.* By O'DONEL BROWNE, M.B., B.Ch., B.A.O., F.R.C.P.I., L.M. (Rotunda Hospital), M.C.O.G. Price 20s.

*The British Journal of Surgery,* XXIII. No. 92. April, 1936. Price 12s. 6d.

*Cystoscopy and Urography.* By J. B. MACALPINE, F.R.C.S. 2nd Edition. Price 30s.

### Local Medical Notes

**The Long Fox Memorial Lecture.**—The twenty-fifth Long Fox Memorial Lecture will be delivered at 8.30 p.m. on Tuesday, 20th October, in the University, by Dr. A. Bulleid, F.S.A. Subject : "Somerset Lake Villages." Admission is free and no tickets are required : but it will be a great convenience if those intending to be present will notify the Secretary, Long Fox Memorial Appointing Board, The University, Bristol.

APPOINTMENTS

**Medical Research Council.**—J. T. Chesterman, F.R.C.S. (Eng.), M.R.C.P. (Lond.), has been appointed to a Travelling Fellowship in Medical Science.

**Bristol Royal Infirmary.**—Assistant Surgeon to Orthopædic Department: K. H. Pridie, M.B., B.S., F.R.C.S. Medical Registrar: M. E. Disney, M.D., M.R.C.P. Surgical Registrar: F. W. Willway, B.Sc., M.D., M.S., F.R.C.S.

